# Correlates of self-harm and suicide attempts in justice-involved young people

**DOI:** 10.1371/journal.pone.0193172

**Published:** 2018-02-15

**Authors:** Stephane Shepherd, Benjamin Spivak, Rohan Borschmann, Stuart A. Kinner, Henning Hachtel

**Affiliations:** 1 Centre for Forensic Behavioural Science, Swinburne University of Technology, Melbourne, Victoria, Australia; 2 Centre for Adolescent Health, Murdoch Children’s Research Institute, Melbourne, Victoria, Australia; 3 Centre for Mental Health, Melbourne School of Population and Global Health, The University of Melbourne, Melbourne, Victoria, Australia; 4 Department of Psychiatry, The University of Melbourne, Melbourne, Victoria, Australia; 5 Section for Women’s Mental Health, Health Service and Population Research Department, Institute of Psychiatry, Psychology & Neuroscience, King’s College London, London, United Kingdom; 6 South London and Maudsley NHS Foundation Trust, London, United Kingdom; 7 Mater Research Institute-UQ, University of Queensland, Brisbane, Queensland, Australia; 8 Griffith Criminology Institute, Griffith University, Brisbane, Queensland, Australia; 9 School of Public Health and Preventive Medicine, Monash University, Melbourne, Victoria, Australia; 10 Netherlands Institute for the Study of Crime and Law Enforcement, Amsterdam, Netherlands; 11 Universitäre Psychiatrische Kliniken (UPK), University of Basel, Basel, Switzerland; Chiba Daigaku, JAPAN

## Abstract

The purpose of this study was to ascertain the prevalence and correlates of self-harm among young people in detention in Australia. The sample included 215 (177 male; 38 female) young people who were in youth detention in the state of Victoria, Australia. Participants were administered a series of questionnaires related to self-harm, mental health, socio-environmental experiences and behaviours. Overall, one-third (33%) of the sample reported previous self-harm and 12% reported at least one suicide attempt. In a multivariate logistic regression analysis, a history of childhood trauma, contact with mental health services, and low educational interest significantly increased the likelihood of self-harm. Young people who reported a suicide attempt scored significantly higher on the measure of childhood trauma than did youth who had engaged in non-suicidal self-harm. Findings demonstrate a strong connection between childhood traumatic experiences and suicidal behaviours for youth in detention. Trauma histories and mental health concerns must be considered when identifying youth at increased risk of self-harm.

## Introduction

Self-harm refers to intentional self-injurious behaviour (such as self-poisoning or self-cutting) with or without suicidal intent [1]. Self-harm is a predictor of future suicide attempts and suicide deaths [2]. In Australia, the median age for death as a result of suicide is around 45 years [3], however the initiation of suicidal behaviour often occurs during adolescence [4,5]. Adolescents who self-harm often have poorer health outcomes and shorter life expectancies [4,6–8]. Moreover, suicide is the leading cause of death among adolescents in Australia [3] and among the leading causes of death internationally [9,10]. As such, suicide and self-harm among young people is a significant public health concern.

Adolescence is a peak period for neuro-developmental change [11]. Self-harming behaviours often commence during this vulnerable stage as it is often characterised by emotional volatility, sensation seeking, risk taking, impulsivity and suggestibility. A number of risk factors have been identified in the literature for youth at-risk for self-harm; these include family breakdown, exposure to trauma, substance misuse, mental ill health, impulsivity, peer ostracism and bullying, victimization, disruption to education, and other negative life events [4,12,13,14]. A number of sub-populations have also been identified as being at an increased risk for self-harm and suicide including Indigenous and LGBT groups [15]. Youth involved in the criminal justice system are one of these sub-populations. Evidence suggests that youth who have had contact with the justice system are more likely to have engaged in self-harm compared to their peers in the general population [16]. In fact, the leading cause of death in youth custodial settings is suicide [17]. Justice-involved youth have often endured cumulative adverse life experiences and present with behavioural and emotional problems [4,16,18–20]. Many of these environmental and clinical concerns are consistent with the risk factors for self-harm.

The bulk of the literature on self-harm and suicide is focused on adults and young people who have not been exposed to the justice system. Research has identified the prevalence and correlates of both in custody and lifetime self-harm among youth justice populations [16,18–23]. However, there have been few studies of the prevalence of self-harm in young people in detention in Australia. Kenny et al. [24] identified recent and lifetime prevalence of self-harm and suicidal ideation for 242 young people in custody in New South Wales (NSW). In this study, 21.9% of the sample had either self-harmed or attempted suicide in the past 12 months. In another study, Indig et al. [25] reported that for 361 young people in custody in NSW, 16.2% had self-harmed and 9.5% had attempted suicide in their lifetime. In a sample of 800 young people serving community-based orders in NSW, 15% of males and 18% of females had self-harmed in the past 12 months [26]. Putnins [27] found that 27% of a sample of 900 young people in detention in South Australia reported a previous suicide attempt. Finally, Borschmann et al. [20] investigated the prevalence and correlates of recent self-harm among 515 young people serving either a community-based order or a custodial sentence in Victoria. In this study, 16.1% had self-harmed in the past six months, with higher rates among those in custody [20].

The objective of the current study was to further increase the knowledge base on the risk factors associated with self-harm for justice-involved young people in Australia. First, we aimed to explore the lifetime prevalence of self-harming behaviours and suicide attempts for the sample. Second, we aimed to identify clinical and environmental correlates of self-harm. Third, we aimed to examine whether the clinical and environmental correlates differentiated between self-harm and suicide attempts for the sample.

## Materials and methods

### Settings

A cohort of young people in custody was recruited from two youth justice centres in the Australian state of Victoria: Parkville Youth Justice Precinct (PYJP) and Malmsbury Youth Justice Centre (MYJC). PYJP accommodates males and females aged 10 to 17 years who have been remanded or sentenced by a Victorian court, and young women aged 18 to 20 years who have been sentenced by a Victorian Court. MYJC accommodates young men aged 18 to 20 years who have been sentenced by a Victorian Court. The inclusion of 18- to 20-year-olds in the youth sample was predicated on Victoria’s “dual track” policy that differentiates people in this age group as being subject to either the adult or youth justice system. This system is reserved for a subset of young adults who are deemed to be particularly impressionable, immature, or likely to be subject to undesirable influences in an adult prison setting, and who have reasonable prospects for rehabilitation [20].

### Measures

#### Childhood Trauma Questionnaire (CTQ)

The CTQ [28] is a self-report instrument that assesses experiences of abuse and neglect in childhood. It is designed to assess five dimensions of childhood maltreatment: physical abuse, emotional abuse, sexual abuse, physical neglect and emotional neglect. Each dimension includes five questions which are individually coded on a 5-point scale (1 = never true to 5 = very often true). Scores from each domain are then summed [5–25] and indicate the level of trauma experienced. The psychometric properties of the CTQ have been extensively validated [28,29,30] and the instrument has been administered with samples of justice-involved young people [26,31,32].

#### Barratt Impulsiveness Scale

The Barratt Impulsiveness Scale (BIS) is a 30-item questionnaire designed to measure the construct of impulsivity [33]. Answers are scored on a 4-point scale (1 = never/rarely to 4 = almost/always). BIS total scores of 72 and above are indicative of a high level of impulsivity. The scale encompasses attentional, motor and non-planning impulsiveness sub-factors. It is one of the most widely employed and extensively validated self-report measures of impulsivity and it is employed regularly in justice-involved populations [33].

#### Welsh Anxiety Inventory

The Welsh Anxiety Inventory (WAI) is a 39-item true/false questionnaire designed to assess anxiety [34]. The measure is derived from the Minnesota Multiphasic Personality Inventory (MMPI) and has demonstrated robust convergent validity [35]. Scores are additive to calculate an overall total, with higher scores indicating a higher degree of self-reported anxiety.

#### Structured Assessment of Violence Risk in Youth

The SAVRY is a structured professional judgment instrument designed to assess risk for violence in young people aged 12–18 years [36]. It comprises 24 risk items across three subscales assessing Historical, Socio/Contextual, and Individual domains.

For the purposes of the present study, six SAVRY items that had shown previous empirical and/or theoretical relationships associations with self-harm were selected to test for an association with self-harm in the sample. These were: a) history of violence; b) stress and poor coping; c) lack of educational interest; d) lack of personal and social support; e) peer rejection; and f) substance use difficulties. Each SAVRY risk factor is coded on a three-point scale (0—Low, 1—Medium, 2—High) which represents the presence and severity of the risk item.

#### Self-harm

Information on self-harming behaviours was compiled from the two sources below.

#### The Victorian Offending Needs Indicator for Youth (VONIY)

The VONIY is an offending needs instrument that provides Victorian youth justice case workers with information for intervention prioritization. At the time of the study, the VONIY was completed internally by youth justice staff via a client interview and the consideration of collateral information (i.e., specialist reports, institutional records). The item ‘self-harm concerns’, which encompassed any history of self-harming behaviours (i.e., suicidal ideation, minor acts of self-mutilation, suicide attempts), was included in this study.

#### SAVRY item 5: History of self-harm or suicide attempts

This item was operationalized across three categories; Low: Youth has no history of self-harm or suicide attempts; Moderate: Youth has a history of self-harm or suicidal actions that did not require medical care and had no clear suicidal intent; High: Youth has a history of medically severe self-harm (requiring medical care or hospitalization) or one or more suicide attempts [36]. This item was scored after a semi-structured interview with each participant.

Based on the above information, four sub-categories of self-harming behaviours were created. These were: ‘No self-harm’ (participants who had no history of self-harm on *both* the SAVRY and the VONIY); ‘Self-harm’ (participants who received a ‘Moderate’ or ‘High’ rating on the SAVRY and/or the presence of self-harm concerns on the VONIY); ‘Non-suicidal self-harm’ (participants who received a ‘Moderate’ rating on the SAVRY only); and ‘Suicide attempts’ (participants who received a ‘High’ rating on the SAVRY only). Non-suicidal self-harm (NSSI) refers to episodes of self-harm where there is no intention of death. VONIY data were not included in the categories ‘Non-suicidal self-harm’ and ‘Suicide Attempts’ as they did not distinguish between these two categories.

#### Mental health contacts

Data on mental health care were obtained from the statewide Redevelopment of Acute & Psychiatric Information Directions (RAPID) database. Data included consenting participants’ mental health records, which comprise a comprehensive summary of every contact and admission to a mental health service (community-based or hospitalisation) in Victoria.

### Procedure

VONIY data were obtained from the Victorian Department of Human Services. The SAVRY and psychological questionnaires were coded by Masters level clinical researchers in the two detention centers. Youth were approached by researchers in custody and asked if they would be interested in hearing about the study. Those who were interested were then invited to have the study explained to them and given the opportunity to ask questions. Written informed consent was obtained from all participants. Consent for participants under 18 years of age fell within the “mature minor” concept as described in Victorian legislation, where mental competency is determined by the ability of an underage participant to understand or appreciate points pertaining to their partaking in, and the nature of, the study. Participants were interviewed individually in private rooms allocated by youth justice custodial center staff. The duration of each assessment was approximately 90 minutes. The consent procedures were approved by the Victorian Department of Human Services Human Research Ethics Committee and the Monash University Human Research Ethics Committee.

### Statistical analysis

Three separate aims were addressed by this study. The first, estimating the prevalence of self-harming behaviours, was addressed by calculating the proportion of participants classified as having attempted suicide and/or engaged in self-harm. Given that the VONIY does not discriminate between self-harm and suicide attempt, whereas the SAVRY does, data are presented separately for each measure. 95% confidence intervals were calculated for the proportions of participants reporting self-harm, suicide attempts or both.

The second aim was to identify factors associated with self-harm or suicide attempts. The aim was addressed through the use of a multivariable logistic regression, in which suicide attempts and self-harm were collapsed to create a binary variable (i.e., no self-harm or suicide attempt vs. self-harm or suicide attempt).

The final aim was to identify factors that differentiated between self-harm and suicide among the sample. Chi-Square tests for independence and associated significance tests were conducted on each individual factor, with an alpha level of .05 adjusted using the Holm-Bonferroni method.

## Results

Data from 215 (*Male* = 177, 82.3%) young people were collected. The mean age of participants was 16.9 (*SD* = 1.9, range: 12–21) years. The sample comprised participants from an English Speaking Background (ESB, 45.1%, n = 105), Culturally and Linguistically Diverse (CALD, 28.8%, n = 67) and Indigenous (18.5%, n = 43) backgrounds. Participants who self-identified as ESB were white and/or Caucasian participants of European descent. Participants who identified as CALD represented minority groups from non-English-speaking backgrounds (e.g., Vietnamese, Sudanese, Pacific Islander, Maori, Lebanese). The Indigenous group comprised participants who self-identified as having Aboriginal Australian or Torres Strait Islander heritages. The majority (89%, *N* = 184) had previously received a police charge for a violent offence and all participants had a self-reported history of violence. The most common index offence was serious assault (31%, *N* = 49).

### Prevalence of self-harm

Prevalence data for each of the outcome assessments used are presented in Table 1. Two participants interviewed by study researchers were excluded from the analysis due to incomplete answers to SAVRY self-harm questions. VONIY data were available for only 181 participants.

**Table 1:** Proportion of participants reporting self-harm behaviour by assessment type.

	n/N	%	(95% CI)
**SAVRY**			
No evidence of NSSI or suicide attempt	165/213	77	71.1–82.8
NSSI	23/213	11	7.1–15.9
Suicide attempt	25/213	12	7.9–17.0
**VONIY**			
No evidence of self-harm	141/181	78	71.0–83.6
Self-harm	40/181	22	16.4–29.0
**VONIY and SAVRY**			
No evidence of self-harm	144/215	67	60.2–73.1
Self-harm	71/215	33	26.8–39.7

Table 1 shows similar proportions of NSSI behaviour (11%) and suicide attempts (12%) in the sample according to the SAVRY. The proportion of participants classified as having displayed self-harm behaviours according to the VONIY (22%) was similar to the proportion of participants classified as displaying either NSSI or suicide attempt according to the SAVRY (23%). The total number assessed as having a history of NSSI or suicide attempts using both measures was considerably larger than the number assessed with each measure alone. Of the 71 participants assessed as having engaged in either NSSI or a suicide attempt, only 17 (24%) were identified as such using both measures.

The gender split within each of the categories was roughly similar between measures, with 19% (n = 29) of males and 36% (n = 11) of females recorded as displaying self-harm behaviours using the VONIY data; and 19% (n = 34) of males and 37% (n = 14) of females recorded as displaying either NSSI or suicide attempt using the SAVRY. When both measures were combined, the gender split indicated that females were more commonly assessed as having engaged in self-harm (n = 20, 53%) compared with males (n = 51, 29%).

### Factors associated with self-harm

A multivariable logistic regression was performed to assess the extent to which each risk factor was associated with reported self-harm. Missing data were treated by listwise removal from the dataset, and in total 40 (18.5%) participants were removed from the model leaving 176 (81.5%). Little’s MCAR test was not significant, χ^2^(8) = 3.64, p = .89, indicating that the pattern of missing data was not inconsistent with a pattern of data missing completely at random.

Multivariable logistic regression operates under the assumption of a linear relationship between continuous variables and the outcome. This assumption was examined with the data through the use of the Box-Tidwell procedure. Significance tests performed on the interaction terms returned p values well in excess of the standard alpha level of .05 (range = .27–.99), suggesting that the data were consistent with a model in which the assumption was valid.

Given the use of continuous variables, the final model did suffer from a considerable sparsity of data, with over 50% of cells containing zero frequencies. As such, calculations of model improvement based on the χ^2^ distribution (e.g., deviance) were not utilised. In addition to the multivariable logistic regression, separate 95% confidence intervals were calculated for each of the categorical and ordinal variables utilised in the model.

The total effect size for the model, evaluated using Nagelkerke’s R^2^, a coefficient which ranges from 0 (equivalent to no predictive power) to 1 (with 1 equivalent to perfect prediction), the R^2^ for the model was 0.51. The predictive power of the model was excellent, with 78.4% of cases correctly classified as either ‘no self-harm’ or ‘self-harm’.

Table 2 presents the correlates of self-harm. Adjusted odds ratios and associated 95% confidence intervals are also presented.

**Table 2:** Correlates of self-harm.

	No Self-harm (N = 118)			Self-harm (N = 58)				
	n	%	(95% CI)	n	%	(95% CI)	OR (95%CI)	AOR (95%CI)^c^
**Continuous measures**^a^								
BIS	-	-	-	-	-	-	1.04 (1.01–1.07)	1.01 (0.97–1.05)
WAI	-	-	-	-	-	-	1.09 (1.05–1.13	1.05 (0.99–1.11)
CTQ	-	-	-	-	-	-	1.06 (1.04–1.08)	1.05*** (1.02–1.08)
**Categorical measures**^b^								
No contact with mental health services	80	67.8	58.5–75.9	19	32.8	21.4–46.5	-	-
Contact with mental health services	38	32.2	24.1–41.5	39	67.2	53.5–78.6	3.36 (1.85–6.07)	3.36** (1.41–8.01)
Low substance use difficulties	18	15.3	9.5–23.3	3	5.2	1.3–15.3	-	-
Mod. substance use difficulties	14	11.9	6.9–19.4	4	6.9	2.2–17.5	1.47 (0.29–7.45)	2.71 (0.33–22.7)
High substance use difficulties	86	72.9	63.8–80.5	51	87.9	76.1–94.6	4.39 (1.26–15.32)	1.92 (0.37–9.96)
Low stress and poor coping	41	34.7	26.4–44.1	8	13.8	6.6–25.9	-	-
Mod. stress and poor coping	39	33.1	24.8–42.4	8	13.8	6.6–25.9	1.44 (0.53–3.97)	0.60 (0.14–2.52)
High stress and poor coping	38	32.2	24.1–41.5	42	72.4	58.9–83.0	7.66 (3.29–17.80)	3.12 (0.95–10.23)
Low peer rejection	58	49.2	39.9–58.5	17	29.3	18.5–42.9	-	-
Mod. peer rejection	40	33.9	25.6–43.3	24	41.4	28.9–55.0	2.45 (1.22–4.89)	2.01 (0.74–5.41)
High peer rejection	20	16.9	10.9–25.2	17	29.3	18.5–42.9	3.69 (1.74–7.84)	1.98 (0.55–7.11)
Low history of violence	12	10.2	5.6–17.4	6	10.3	4.3–21.8	-	-
Mod. history of violence	19	16.1	10.2–24.3	4	6.9	2.2–17.5	0.43 (0.12–1.54)	0.57 (0.14–2.52)
High history of violence	87	73.7	64.7–81.2	48	82.8	70.1–91.0	1.05 (0.40–2.77)	0.38 (0.09–3.60)
Low lack of educational interest	53	44.9	35.8–54.3	10	17.2	9.0–29.9	-	-
Mod. lack of educational interest	25	21.2	14.4–29.9	19	32.8	21.4–46.5	2.58 (1.17–5.66)	3.92* (1.05–14.6)
High lack of educational interest	40	33.9	25.6–43.3	29	50.0	37.5–62.5	3.14 (1.51–6.50)	1.81 (0.59–5.55)

^a^ Proportional statistics are not provided for continuous measures

^b^ Reference category for categorical measures is low or absent (e.g. absence of contact with mental health services).

^c^ Odds ratios are adjusted for all variables listed in the model.

*p<.05;

**p<.01,

***p<.001

The results presented in Table 2 indicate that three factors were significantly associated with self-harm: total score on the Childhood Trauma Questionnaire (OR = 1.04 per unit change in CTQ score, 95%CI = 1.01–1.07); Thus, the adjusted odds ratio for the maximum score on the CTQ (125) relative to 0 is equal to OR = 577, 95%CI = 17.6–18965; contact with mental health services; and having a moderate lack of educational interest. Specifically, a moderate lack of educational interest was associated with a significant increase in predictive power relative to low lack of educational interest.

### Factors differentiating between NSSI and suicide attempts

Given the small number of participants who were reported to have self-harmed (n = 23) or attempted suicide (n = 25) on the SAVRY, the use of a regression model was not considered a valid approach to analysis. Chi-Square tests for independence and associated significance tests were conducted on each individual factor, with an alpha level of .05 adjusted using the Holm-Bonferroni method to correct for type I error inflation.

Three t-tests were conducted to test for mean differences between participants assessed as having engaged only in NSSI (n = 23) and participants assessed as having attempted suicide (n = 25). The first t-test examined the average total score on the Barratt Impulsiveness Scale and found no significant difference between participants displaying NSSI (M = 81, SD = 12.4) and participants who had attempted suicide (M = 81.7, SD = 13.2), t(38) = -0.16, p = .87, d = 0.05. Similarly, a t-test comparing total scores on the Welsh Anxiety Inventory found no significant differences between the NSSI group (M = 24.1, SD = 9.29) and the suicide attempt group (M = 23.7, SD = 7.69), t(43) = 0.14, p = .89, d = 0.04. However, a t-test comparing total scores on the Childhood Trauma Questionnaire between the NSSI group (M = 50.8, SD = 16.3) and the suicide attempt group (M = 71.6, SD = 26.0) did reveal a significant difference; t(40) = -2.9, p = .005, d = 0.95.

Chi-square tests of independence were conducted for each of the categorical risk factors across the two self-harm categories (NSSI and Suicide attempts). Results are displayed in Table 3.

**Table 3:** Risk factor distributions across self-harm categories.

Risk factors	NSSI N = 23	Suicide attempt N = 25	Χ^2^	*p*	V
No contact with mental health services	15	7	-	-	-
Contact with mental health services	8	18	6.68	.06	0.26
Low peer rejection	5	3	-	-	-
Moderate peer rejection	12	6	-	-	-
High peer rejection	6	16	6.97	.15	0.26
Low stress and poor coping	1	2	-	-	-
Moderate stress and poor coping	5	1	-	-	-
High stress and poor coping	17	22	3.56	.39	0.19
Low substance use issues	0	2	-	-	-
Moderate substance use issues	2	0	-	-	-
High substance use issues	21	23	4.02	.39	0.20
Low history of violence	4	1	-	-	-
Moderate history of violence	3	0	-	-	-
High history of violence	16	24	6.33	.16	0.25
Low lack of educational interest	8	3	-	-	-
Moderate lack of educational interest	5	9	-	-	-
High lack of educational interest	10	13	3.73	.39	0.19

None of the results reported were significantly different from what would be expected on the basis of chance alone. All effect sizes were small in magnitude.

## Discussion

This study ascertained the prevalence and correlates of self-harming behaviours in a sample of young people in custody. Approximately one-third (33%) of the sample had engaged in previous self-harm. Where it could be verified, at least 11% of the participants had engaged in non-suicidal self-injury and a further 12% had attempted suicide. The strongest correlates of self-harm were childhood trauma, prior contact with mental health services, and a moderate lack of educational interest. Participants who had attempted suicide reported more severe histories of childhood trauma compared to participants who had engaged in non-suicidal self-injury only.

The lifetime prevalence of self-harming behaviours in the sample fell within the range identified in the international literature for youth in custody (approximately 10–40%) [16,19]. The proportion of life-time self-harm is somewhat higher than rates identified in previous Australian research [25]. However, the bulk of these studies reported self-harm activity within a specific period of time (i.e., past 6–12 months) [20,24,26]. Either way, the prevalence of self-harm identified in this study, with regard to the extant international and regional literature, is somewhat elevated. In some ways, this could be explained by the traditional welfarist nature of the Victorian youth justice system. A focus on youth diversionary alternatives to custody has generally resulted in comparatively lower numbers of young people in custody in Victoria compared to other Australian states [37,38], yet those who are detained are often at the severe end of the offending spectrum and present with multiple complex health and social needs. Adverse life experiences are commonplace among Victorian youth in custody [39,40] and are similarly reported to pre-date [4] and follow [6,7] self-harming behaviours. The greater prevalence of self-harming behaviours among females in the sample was consistent with prior literature [5,19,20,25,26].

Childhood experiences of trauma were among the strongest correlates of self-harm in the sample. There was a striking increase in the likelihood of self-harm for youth who recorded upper range scores on the CTQ versus youth who scored in the lower ranges. Childhood trauma was also found to be more prevalent among youth who reported an attempted suicide compared to those who reported NSSI only. A number of studies have identified various forms of child maltreatment as predicting self-harm/suicide attempts [41–46] in the general population, as well as predicting self-harming behaviours in custody [19,23,24,47]. Childhood trauma is also associated with youth suicide in custody [48]. Elevated levels of trauma are common in youth correctional samples [30,49]. Mental illness is also disproportionately higher in such populations [25,50,51], and is often associated with traumatic experiences [52–54]. In this study, contact with statewide public mental health services was a strong correlate of self-harm. It is unknown whether study participants visited or were admitted to public mental health services specifically because of self-harming concerns. However it is possible that youth who had contact with mental health services were more likely to have been in crisis at that particular time. Prior research has consistently demonstrated robust associations between psychiatric symptoms and self-harm among a) justice-involved young people [16,20,22,24,27,55]; b) those accessing clinical mental health services [56,57] and c) general population samples [58–60]. A link between self-harm during adolescence and later mental health problems has also been established [6,7]. The notable connection between childhood trauma, mental illness and self-harm underscores the vulnerability of this custodial sample, signifying the importance of therapeutic approaches to custodial care and management.

A significant association was also identified between a ‘moderate’ lack of educational interest and self-harm. A ‘moderate’ rating specifically refers to youth who attend school regularly but have little interest in, or commitment to, school. This differs from a ‘high’ rating which refers to youth who are both uninterested in school and are regularly truant. It is unknown why ‘moderately’ interested youth specifically are particularly vulnerable to self-harm in this sample, whilst those with a ‘high’ rating are not. Perhaps regular attendance in an activity in which one is uninterested, or an environment in which one is uncomfortable being (perhaps from bullying/peer rejection/learning difficulties) may engender distress. In contrast, youth who presented with a ‘high’ lack of educational interest may be less exposed to school related stressors as they do not attend regularly or have disengaged from education entirely.

### Clinical implications

Justice-involved young people often have complex health and social needs which require supports within correctional systems and beyond [25,32,39]. Youth detention centres should have protocols in place to identify and manage youth who are at-risk for self-harm. This may include screening during intake, during periods of distress and on release. Screening should also include a detailed history of any traumatic experiences endured. Any young person who is identified as being at increased risk for self-harm should be considered for immediate psychological intervention and a continuing support plan [61]. These supports must be sustained during transition back to the community, which is a particularly vulnerable time for self-harm and suicide risk [62]. It is recommended that there should be correctional workplace training for self-harm and suicide intervention. Youth workers must be able to identify both the psychological/behavioural and physical signs of self-harm and have in place subsequent case management procedures, including prompt referral to psychological supports. An enhanced institutional understanding of the high prevalence of traumatic experiences among youth justice clientele is needed. This includes an awareness of how custodial environments can exacerbate trauma symptoms or re-traumatize clients through institutional practices (i.e., segregation, invasive personal searches). A safer custodial environment with trauma-informed workers may help reduce distress and risk for self-harm.

The study has a number of strengths. It is one of the few studies internationally to ascertain both the prevalence and correlates of self-harm in a youth custodial sample. Furthermore, the study uniquely investigated differences in the prevalence of risk factors for self-harm across different categories of self-harm. The study also had a number of potential limitations. First, the definition of self-harm was wide-ranging after combining both VONIY and SAVRY data. The VONIY data were inclusive of suicidal ideation which does not fall within typical definitions of self-harm. Moreover, the use of two self-harm sources may have contributed to the higher-than-average number of participants who reported self-harm. Alternatively, the use of two sources perhaps allowed for an improved detection of self-harm. Moreover, risk for suicide is often higher in samples with severe offending histories [56]. Second, the cross-sectional nature of the dataset precluded ascertaining any causal, directional or temporal relationships. The analysis was unable to determine whether self-harming occurred prior to, or during, the custodial sentence. Finally, although mental health contacts were predictive of self-harm, the prevalence of psychiatric disorder in the sample may be underestimated, given the reluctance to diagnose young people under the age of 18 with a personality disorder (and the established link between self-harm and personality disorder, particularly borderline personality disorder, [63]).

### Conclusion

Identifying the prevalence and correlates of self-harm for young people in detention is an important public health endeavour. Such information can help correctional services better identify at-risk youth and facilitate therapeutic assistance. Moreover it is important that ongoing self-harm support services are available for at-risk young people leaving custody. This study found that a significant minority of young people in custody have self-harmed and that childhood trauma and mental health problems were linked with these behaviours. Correctional services must be aware of the trauma histories of justice-involved young people and the impact these experiences exert on their mental health and risk for self-harm.
